# Clinical Profile, Comorbidities, and Antimicrobial Resistance Patterns of Myroides Species Isolated From Nosocomial Urinary Tract Infections at a Tertiary Care Center in India: A Retrospective Observational Study

**DOI:** 10.7759/cureus.110211

**Published:** 2026-06-03

**Authors:** Pratiksha Kamboj, Gaurav Badoni, Vanya Singh, Ranjana Rohilla, Binal Mangroliya, Vikas K Panwar, Balram Omar, Pratima Gupta

**Affiliations:** 1 Microbiology, All India Institute of Medical Sciences, Rishikesh, Rishikesh, IND; 2 Urology, All India Institute of Medical Sciences, Rishikesh, Rishikesh, IND

**Keywords:** antimicrobial susceptibility, minocycline, multidrug resistance, myroides species, nosocomial infection, opportunistic infection, urinary tract infection

## Abstract

Background

*Myroides *species are gram-negative, aerobic bacilli within the family Flavobacteriaceae that have emerged as clinically significant, multidrug-resistant nosocomial pathogens. Although historically regarded as organisms of low pathogenicity, they are increasingly associated with opportunistic infections, particularly urinary tract infections (UTIs), among hospitalized, immunocompromised, and catheterized patients. Reports of *Myroides* infections have increased in recent years across different geographical regions, highlighting its emerging global clinical significance. The incidence of reported *Myroides* infections has increased steadily over the last two decades and is recognized as an opportunistic pathogen associated with multidrug resistance. Limited information is available on their clinical characteristics and antimicrobial susceptibility patterns from the Indian subcontinent. Hence, this study aimed to characterize the demographic, clinical features, and antimicrobial resistance patterns of *Myroides* isolates recovered from nosocomial UTIs.

Methodology

This retrospective observational study was conducted over a 6.5-year period (January 2019-July 2025). All consecutive urine culture isolates producing *Myroides* spp. at significant colony counts (≥10^5^ CFU/mL) were included. Isolate identification and antimicrobial susceptibility testing (AST) were performed using VITEK® 2 Compact 60 (bioMérieux, France) or MicroScan WalkAway Model DxM 1096 (Beckman Coulter, Inc., United States). Demographic data, clinical characteristics, risk factors, and antimicrobial susceptibility patterns were examined.

Results

A total of 53 *Myroides* isolates were identified. The median age of the patients was 43.5 years (interquartile range = 30-57), with a predominance of males (33, 62.3%). The majority of the cases were from Urology (17, 32.1%) and General Medicine (11, 20.8%) departments. Device in situ (46, 86.8%) and immunocompromised state (20, 37.7%) were the most common predisposing factors. Mortality was observed in (13, 24.5%) patients, with non-survivors more commonly being male and immunocompromised. Minocycline was the only antibiotic that showed (38/38, 100%) susceptibility. All isolates showed resistance to aminoglycosides (amikacin, gentamicin), fluoroquinolones (ciprofloxacin, levofloxacin), and the majority of the β-lactams tested. Trimethoprim-sulfamethoxazole was susceptible in only 8 (15.1%) isolates.

Conclusions

*Myroides *spp. is a significant but frequently overlooked nosocomial threat, characterized by extensive multidrug resistance and often isolated in high-dependency inpatient settings. Minocycline consistently retains antimicrobial activity against *Myroides *spp. Accurate species identification and systematic AST reporting are important for appropriate clinical management.

## Introduction

*Myroides* spp. are aerobic, non-motile, Gram-negative bacilli classified under the family Flavobacteriaceae, phylum Bacteroidota. The genus was officially established by Vancanneyt and colleagues in 1996 following reclassification of Flavobacterium odoratum Stutzer 1929, with *Myroides odoratus *and *Myroides odoratimimus* identified as the two principal species of clinical importance [1,2]. Historically regarded as environmental commensals of low virulence, *Myroides *spp. are now increasingly regarded as opportunistic nosocomial pathogens, particularly in immunocompromised or device-dependent hosts [3-7].

The clinical significance of *Myroides *spp. lies primarily in their propensity to cause urinary tract infections (UTIs), bloodstream infections, wound infections, and soft-tissue infections, particularly during prolonged hospital stays, with indwelling urinary catheters, or in the presence of underlying immune deficiency [4-7]. A characteristic feature of this genus is its intrinsic and acquired resistance to multiple clinically relevant antibiotics, including all β-lactams, aminoglycosides, and fluoroquinolones, through mechanisms such as metallo-β-lactamases, efflux pumps, and porin modification [8]. This pan-resistance profile significantly limits therapeutic options and is associated with treatment failure and mortality.

Despite the increasing number of *Myroides*-associated infections reported worldwide, systematically collected data describing the clinical epidemiology, host risk factors, outcomes, and antimicrobial susceptibility patterns of *Myroides* UTIs remain limited. The available literature is largely restricted to individual case reports and small case series, which have highlighted the organism’s multidrug-resistant phenotype, susceptibility to minocycline, and occurrence in critically ill or healthcare-exposed patients [9,10]. However, these reports have not quantified the clinical outcomes, departmental distribution, burden of infection, or spectrum of predisposing host factors at length. This knowledge gap limits the ability to define disease burden, inform empiric and targeted therapy, and develop evidence-based infection prevention strategies [9,10].

The present study addresses these gaps by describing the clinical profile, comorbidity burden, risk factor distribution, outcomes, and in vitro antimicrobial susceptibility patterns of *Myroides* isolates recovered from nosocomial UTIs over a 6.5-year period at a tertiary care center in India.

## Materials and methods

Study period

The present retrospective observational study was conducted at a tertiary care teaching hospital in India. The study period spanned from January 2019 to July 2025. However, processing of urine samples in the microbiology laboratory was temporarily suspended for approximately 18 months, from 2021 to July 2022, during the second phase of the COVID-19 pandemic. This interruption was accounted for while interpreting the data. Ethical approval was obtained from the Institutional Ethics Committee (approval number: AIIMS/IEC/26/165) before data collection. The study adhered to the principles of the Declaration of Helsinki.

Patient selection

All patients from whom urine cultures yielded *Myroides *spp. at a significant colony count of ≥10^5^ CFU/mL during the study period were considered for inclusion. Exclusion criteria were (i) colony count <10^5^ CFU/mL; (ii) polymicrobial growth with ≥3 organisms; (iii) incomplete clinical or microbiological records; and (iv) outpatient cultures. Thus, isolates from patients with community-acquired UTI, insignificant colony counts, mixed growth suggestive of contamination, incomplete essential clinical data, or colonization without clinical evidence of UTI were excluded.

Study objective

This study aimed to describe the demographic and clinical profile, predisposing risk factors, and phenotypic antimicrobial resistance patterns of laboratory-confirmed *Myroides* spp. isolated from nosocomial UTIs at a tertiary care teaching hospital in India over a 6.5-year period.

Microbiological identification

Urine samples were processed using standard microbiological techniques. Isolate identification and susceptibility were performed using the VITEK® 2 (bioMérieux, France) and the MicroScan WalkAway Plus (Beckman Coulter, Inc., USA) automated systems. Species-level identification required a concordant result from both platforms. Antimicrobial susceptibility testing (AST) was performed using a panel of antimicrobial agents, with interpretation based on Clinical and Laboratory Standards Institute (CLSI) breakpoints where available, with supplementary reference to European Committee on Antimicrobial Susceptibility Testing (EUCAST) guidance for agents that lacked CLSI-defined breakpoints for *Myroides *spp.

Data collection

Demographic, clinical, and outcome data were extracted from electronic medical records by trained investigators using a standardized data collection form. Variables recorded included age, sex, specimen type, predisposing host factors (presence of indwelling urinary device, immunocompromised state, urinary obstruction), primary diagnosis, comorbidities, and clinical outcome (discharged alive, died in hospital, or left against medical advice (LAMA)).

Definitions

Nosocomial UTI was defined as a UTI diagnosed ≥48 hours after hospital admission with no evidence of infection at admission. Immunocompromised state was defined as the presence of hematological malignancy, solid organ malignancy on active therapy, HIV infection, organ transplantation, or receipt of high-dose corticosteroids or other immunosuppressive agents. Device in situ included indwelling per urethral urinary catheter, percutaneous nephrostomy (PCN) tube, or ureteric double-J stent (DJ stent). Multidrug resistance (MDR) was defined per the Centers for Disease Control and Prevention (CDC) criteria as non-susceptibility to at least one agent in three or more antimicrobial categories.

Statistical analysis

Data analysis was performed using SPSS Statistics version 26.0 (IBM Corp., Armonk, NY, USA). Continuous variables were presented as mean ± standard deviation (SD) or median with interquartile range (IQR), as appropriate. Categorical variables were described as frequencies and percentages. Comparisons between survivors and non-survivors were performed using the chi-squared test or Fisher’s exact test for categorical variables, and the independent samples t-test or Mann-Whitney U test for continuous variables. A two-tailed p-value <0.05 was considered statistically significant.

## Results

Patient demographics and clinical characteristics

Over the 6.5-year study period, 53 non-duplicate *Myroides *spp. isolates from urine cultures met the inclusion criteria. The demographic and clinical characteristics of the study population are summarized in Table 1. The overall study population had a mean age of 44.30 ± 16.91 years. Males constituted the majority (33, 62.3%). Urology (17, 32.1%) and General Medicine (11, 20.8%) departments contributed the highest case burden, collectively accounting for more than half of all isolates. Other departments included Emergency Medicine (6, 11.3%), Neurology/Neurosurgery (4, 7.5%), Trauma (3, 5.7%), Obstetrics & Gynecology (2, 3.8%), and Surgical Intensive Care Unit/High-Dependency Unit (2, 3.8%). Clinical specimens were predominantly urinary, with urine accounting for 50/53 (94.3%) and PCN fluid specimens contributing 3/53 (5.7%).

**Table 1:** Demographic and clinical characteristics of patients with Myroides spp. urinary tract infection (n = 53).

Variable	n (%) or mean ± SD
Age (years), mean ± SD (range)	44.30 ± 16.91 (17–80)
Median age (IQR)	43.5 (30–57)
Sex	
Male	33 (62.3%)
Female	20 (37.7%)
Department	
Urology	17 (32.1%)
General Medicine	11 (20.8%)
Emergency	6 (11.3%)
Neurology/Neurosurgery	4 (7.5%)
Trauma	3 (5.7%)
Others	12 (22.6%)
Predisposing factors	
Device in situ	46 (86.8%)
Immunocompromised state	20 (37.7%)
Urinary obstruction	10 (18.9%)
Comorbidities	
Diabetes mellitus	7 (13.2%)
Malignancy (any)	7 (13.2%)
Hypertension	5 (9.4%)
Chronic liver disease	3 (5.7%)
Other (HIV, chronic kidney disease, tuberculosis)	3 (5.7%)
Clinical outcome	
Discharged alive	33 (62.3%)
In-hospital death	13 (24.5%)
Left against medical advice	7 (13.2%)

Predisposing risk factors and comorbidities

The major predisposing risk factor was the presence of an indwelling urinary device (46, 86.8%), followed by immunocompromised state (20, 37.7%), and urinary tract obstruction (10, 18.9%). Comorbidities were present in a substantial proportion of patients. Diabetes mellitus and active malignancy were the most frequent comorbidities (each 7, 13.2%), followed by systemic hypertension (5, 9.4%), chronic liver disease (3, 5.7%), and HIV infection, chronic kidney disease, and tuberculosis in individual patients (Table 1). Regarding association with coronary artery disease (CAD), 4/4 (100%) deaths versus 9/49 (18.4%) deaths without CAD (Fisher’s p ≈ 0.0024) were noted; however, due to the small sample size, this could not be considered as a single predictor of disease.

Clinical outcomes

Table 2 presents the clinical characteristics of 53 *Myroides* spp. isolates categorized according to patient outcomes (alive: N = 33; death: N = 13; LAMA: N = 7). There was no significant difference in outcomes across different age groups (p = 0.3). Although males were particularly overrepresented among deaths (11, 84.7%), this did not reach statistical significance (p = 0.2).

**Table 2:** Patient characteristics by outcome. P-value = Kruskal-Wallis rank-sum test (H); Fisher’s exact test (χ²); df = degree of freedom; LAMA = left against medical advise; PCN = percutaneous nephrostomy

Characteristic	Overall (N = 53), n (%)	Alive (N = 33), n (%)	Death (N = 13), n(%)	LAMA (N = 7), n (%)	Test value (df)	P-value
Age (years), mean ± SD	44.30 ± 16.91	42.27 ± 17.60	49.62 ± 15.25	44.00 ± 16.62	H = 2.193 (2)	0.3
Sex					χ² = 3.679	0.2
Female	20 (37.7%)	15 (45.45 %)	2 (15.38%)	3 (42.85%)		
Male	33 (62.3%)	18 (54.54%)	11 (84.61%)	4 (57.14%)		
Specimen type					χ² = 0.530	>0.9
PCN	3 (5.66%)	2 (6.06%)	1 (7.7%)	0 (0%)		
Urine	50 (94.3%)	31 (93.9%)	12 (91.9%)	7 (100.0%)		
Device in situ					χ² = 14.254	0.003
Absent	7 (13.2%)	3 (9.1%)	0 (0%)	4 (57.1%)		
Present	46 (86.8%)	30 (90.9%)	13 (100%)	3 (42.9%)		
Immunocompromised state					χ² = 4.096	0.13
Absent	33 (62.3%)	24 (72.7%)	6 (46.2%)	3 (42.9%)		
Present	20 (37.7%)	9 (27.3%)	7 (53.8%)	4 (57.1%)		
Age group (years)					χ² = 6.134	0.3
≤20	3 (5.7%)	2 (6.1%)	0 (0%)	1 (14.3%)		
21–40	22 (41.5%)	17 (51.5%)	3 (23.1%)	2 (28.6%)		
41–60	19 (35.8%)	9 (27.2%)	7 (53.8%)	3 (42.9%)		
61–80	9 (16.9%)	5 (15.15%)	3 (23.1%)	1 (13.9%)		
>80	0 (0%)	0 (0%)	0 (0%)	0 (0%)		

Two variables showed statistically significant associations with outcomes. Device in situ was significantly associated with outcome (p = 0.003). All 13 patients who died had a device present (100%), suggesting that indwelling devices may have played an important role in mortality in *Myroides* infections.

Immunocompromised status, though not statistically significant (p = 0.13), showed a clinically noteworthy trend being present in seven (53.8%) deaths and four (57.1%) LAMA patients compared to only 27% of survivors, warranting consideration in larger studies. The 41-60-year age group accounted for the largest proportion of deaths (7, 53.8%), though the overall age group distribution did not differ significantly across outcomes (p = 0.3) (Table 2).

Antimicrobial susceptibility profile

All 53 isolates were identified using an automated identification system. The susceptibility results are presented in Table 3. Minocycline demonstrated the highest in vitro activity, with 100% (38/38) susceptibility among those tested. All tested β-lactams, including cefotaxime, ceftazidime, cefepime, and piperacillin-tazobactam, demonstrated very low efficacy, with susceptibility rates below 4%. Ticarcillin-clavulanic acid showed complete resistance. Aminoglycosides (amikacin, gentamicin, tobramycin) and fluoroquinolones (ciprofloxacin, levofloxacin) demonstrated 100% resistance across all tested isolates. Trimethoprim-sulfamethoxazole (co-trimoxazole) exhibited limited activity, with susceptibility in 8/53 isolates (15.1%) and intermediate susceptibility in 1/53 (1.9%). Tetracycline susceptibility was similarly low (<4%). All isolates fulfilled criteria for MDR (Table 3).

**Table 3:** Antimicrobial susceptibility profile of Myroides spp. isolates (n = 53 unless stated). Susceptibility interpreted per Clinical and Laboratory Standards Institute (CLSI) breakpoints. The European Committee on Antimicrobial Susceptibility Testing guidance was applied where CLSI breakpoints were unavailable for *Myroides *spp.

Antibiotic	Class	Susceptible, n (%)	Resistant, n (%)	Notes
Minocycline	Tetracycline	38/38 (100%)	0 (0%)	n = 38
Trimethoprim–sulfamethoxazole	Folate inhibitor	8 (15.1%)	44 (83.0%)	1 intermediate
Piperacillin-tazobactam	β-lactam/β-lactamase inhibitor	<4%	≥96%	-
Ceftazidime	Cephalosporin	<4%	≥96%	-
Cefepime	Cephalosporin	<4%	≥96%	-
Cefotaxime	Cephalosporin	0 (0%)	53 (100%)	-
Ticarcillin-clavulanic acid	β-lactam/β-lactamase inhibitor	0 (0%)	53 (100%)	-
Tetracycline	Tetracycline	<4%	≥96%	-
Amikacin	Aminoglycoside	0 (0%)	53 (100%)	-
Gentamicin	Aminoglycoside	0 (0%)	53 (100%)	-
Tobramycin	Aminoglycoside	<4%	≥96%	-
Ciprofloxacin	Fluoroquinolone	0 (0%)	53 (100%)	-
Levofloxacin	Fluoroquinolone	0 (0%)	53 (100%)	-

## Discussion

This study depicts one of the largest single-center retrospective analyses of *Myroides *spp. UTIs from an Indian tertiary care setting. Our findings show that *Myroides *spp. are emergent nosocomial infections with predictable grouping in high-dependency inpatient settings, strong epidemiological links to device use and immunosuppression, and a near-universal MDR phenotype that severely restricts therapeutic options.

The predominance of Urology and General Medicine as the source departments is consistent with prior reports from India [9,10] and demonstrates the occurrence of *Myroides spp.* within hospital environments. Urological patients are excessively represented due to the high frequency of indwelling urinary devices such as nephrostomy tubes, DJ stents, and Foley’s catheters, which serve as niduses for biofilm formation. *Myroides *spp. possess strong biofilm-forming capability [11,12], which enables prolonged persistence on device surfaces while impairing penetration, and predisposes to recurrent or refractory infection. The presence of device in-situ and obstruction was statistically significant in our cohort and is consistent with the proposed pathogenetic mechanism.

The high proportion of urinary specimens and nephrostomy-related samples is consistent with a clinical ecology in which colonization or infection is facilitated by urinary tract instrumentation, obstruction, and repeated healthcare contact, all of which can increase exposure to hospital flora and selective antibiotic pressure. From a practice perspective, this matters because it suggests that the preventive strategies should focus more on device stewardship, catheter/nephrostomy care bundles, and early source control in at-risk patients.

Although immunocompromised status was not statistically significant, the observed trend toward higher mortality in immunocompromised patients aligns with previous case series [13] and supports the hypothesis of *Myroides *spp. as an opportunistic pathogen.

The antimicrobial susceptibility data confirm the pan-resistant phenotype of *Myroides *spp. Complete resistance to all tested aminoglycosides and fluoroquinolones, and near-complete resistance to all β-lactams, is linked to the intrinsic production of metallo-β-lactamases (which hydrolyze carbapenem and other β-lactam scaffolds), active efflux pump systems (primarily of the resistance-nodulation-division family), and outer membrane porin modifications that reduce intracellular antibiotic accumulation [8]. Minocycline demonstrated 100% susceptibility, concordant with prior Indian reports [9,10,14] and remains the treatment of choice based on in vitro data. The low co-trimoxazole susceptibility rate (15.1%) in our cohort varies from the 100% resistance reported by Savci et al. [15] and underscores the geographic and temporal variability in resistance patterns, potentially attributed to differences in antibiotic selection pressures. Therefore, clinicians should avoid assuming co-trimoxazole susceptibility without organism-specific AST.

From a diagnostic perspective, accurate species-level identification of *Myroides *spp. requires mass spectrometric (matrix-assisted laser desorption/ionization time-of-flight) or automated methods. Conventional biochemical identification systems may misidentify or fail to speciate this genus. Timely and accurate identification is essential to ensure that appropriate targeted therapy guided by AST is initiated promptly, particularly given the inability to rely on empirical agents conventionally used for Gram-negative UTIs.

This study has certain limitations. First, the single-center retrospective design introduces potential selection and ascertainment bias. Second, species-level differentiation was not consistently documented, excluding species-specific outcome analysis. Species identification relied on automated platforms without molecular confirmation. Third, the antibiogram panel of 16 antibiotics did not include colistin, fosfomycin, or rifampicin agents with limited activity against *Myroides *spp. reported in the literature, limiting the therapeutic options that could be formally assessed. Fourth, the absence of formal multivariable outcome modelling, attributable to sample size constraints, hampers causal interpretation of mortality-associated risk factors. Lastly, the LAMA group (13.2%) introduces outcome misclassification bias if some of these patients subsequently died outside the hospital. Fifth, the inability to definitively distinguish catheter-associated UTI from asymptomatic colonization in all catheterized patients. Due to the retrospective design, positive urine cultures could not always be correlated with clinical evidence of infection.

Future prospective multicenter studies with standardized AST panels including novel agents, molecular epidemiological typing, and robust multivariable outcome analyses are warranted.

## Conclusions

*Myroides *spp. are emerging MDR nosocomial pathogens causing UTIs predominantly in catheterized and immunocompromised inpatients, with a particular burden in urology and critical care settings. The in-hospital mortality in this cohort highlights their clinical significance. Minocycline was the only consistently active antimicrobial agent in vitro and may represent an option for targeted therapy of UTIs caused by *Myroides *spp. In our study, the isolates demonstrated extensive resistance to commonly used empirical agents for Gram-negative UTIs, including β-lactams, fluoroquinolones, and aminoglycosides, highlighting the limitations of standard empirical regimens against this pathogen. Accurate species-level identification using automated methods for identification, coupled with routine AST and timely reporting, is essential to guide appropriate therapy and optimize clinical management.

## References

[1] Stutzer MJ (1923). Zur frage über die fäulnisbakterien im darm. Zentralbl Bakteriol Parasitenkd Infekt Hyg.

[2] Vancanneyt M, Segers P, Torck U (1996). Reclassification of Flavobacterium odoratum (Stutzer 1929) strains to a new genus, Myroides, as Myroides odoratus comb nov and Myroides odoratimimus sp nov.. Int J Syst Bacteriol.

[3] Agrawal M, Mamoria VP, Mittal S, Sharma A (2019). Myroides: an emerging pathogen causing urinary tract infections in hospitalized patients. Int J Contemp Med Res.

[4] Benedetti P, Rassu M, Pavan G, Sefton A, Pellizzer G (2011). Septic shock, pneumonia, and soft tissue infection due to Myroides odoratimimus: report of a case and review of Myroides infections. Infection.

[5] Ktari S, Mnif B, Koubaa M, Mahjoubi F, Ben Jemaa M, Mhiri MN, Hammami A (2012). Nosocomial outbreak of Myroides odoratimimus urinary tract infection in a Tunisian hospital. J Hosp Infect.

[6] Endicott-Yazdani TR, Dhiman N, Benavides R, Spak CW (2015). Myroides odoratimimus bacteremia in a diabetic patient. Proc (Bayl Univ Med Cent).

[7] Lorenzin G, Piccinelli G, Carlassara L, Scolari F, Caccuri F, Caruso A, De Francesco MA (2018). Myroides odoratimimus urinary tract infection in an immunocompromised patient: an emerging multidrug-resistant micro-organism. Antimicrob Resist Infect Control.

[8] Hu SH, Yuan SX, Qu H, Jiang T, Zhou YJ, Wang MX, Ming DS (2016). Antibiotic resistance mechanisms of Myroides sp. J Zhejiang Univ Sci B.

[9] Sahu C, Chaudhary R, Bhartiya C, Patel SS, Bhatnagar N (2024). A retrospective study on UTI by Myroides species: an emerging drug resistant nosocomial pathogen. Indian J Crit Care Med.

[10] Mahendran AJ, Agrawal S, Rastogi N, Gupta N (2021). Myroides: a rare but hard-to-crack villain in a critical care setup. Indian J Crit Care Med.

[11] Pompilio A, Galardi G, Gherardi G (2018). Infection of recurrent calcaneal ulcer caused by a biofilm-producer Myroides odoratimimus strain. Folia Microbiol (Praha).

[12] Lu Y, Xia W, Zhang X, Ni F, Mei Y (2020). A confirmed catheter-related blood stream infection (CRBSI) in an immunocompetent patient due to Myroides odoratimimus: case report and literature review. Infect Drug Resist.

[13] Chauhan K, Chaturvedi P, Singh RP, Pandey A (2020). Myroides causing catheter associated urinary tract infection in diabetic patients: an emerging multidrug resistant 'superbug'. J Clin Diagn Res.

[14] Khan U, Pandey E, Gandham N (2023). A case series and literature review of infections due to Myroides spp.: identification of contributing factors and emerging antibiotic susceptibility trends. Access Microbiol.

[15] Savci U, Eser B, Sungur M, Yildiz SS, Akdogan O, Caliskan S, Sahin M (2025). A new emerging threat in critical care: Myroides odoratimimus outbreak in a tertiary hospital. Indian J Crit Care Med.

